# Inhibition of homologous phosphorolytic ribonucleases by citrate may represent an evolutionarily conserved communicative link between RNA degradation and central metabolism

**DOI:** 10.1093/nar/gkx114

**Published:** 2017-03-03

**Authors:** Carlanne M. Stone, Louise E. Butt, Joshua C. Bufton, Daniel C. Lourenco, Darren M. Gowers, Andrew R. Pickford, Paul A. Cox, Helen A. Vincent, Anastasia J. Callaghan

**Affiliations:** 1 School of Biological Sciences and Institute of Biomedical and Biomolecular Sciences, University of Portsmouth, Portsmouth, PO1 2DY, UK; 2 School of Pharmacy and Biomedical Sciences and Institute of Biomedical and Biomolecular Sciences, University of Portsmouth, Portsmouth, PO1 2DT, UK

## Abstract

Ribonucleases play essential roles in all aspects of RNA metabolism, including the coordination of post-transcriptional gene regulation that allows organisms to respond to internal changes and environmental stimuli. However, as inherently destructive enzymes, their activity must be carefully controlled. Recent research exemplifies the repertoire of regulatory strategies employed by ribonucleases. The activity of the phosphorolytic exoribonuclease, polynucleotide phosphorylase (PNPase), has previously been shown to be modulated by the Krebs cycle metabolite citrate in *Escherichia coli*. Here, we provide evidence for the existence of citrate-mediated inhibition of ribonucleases in all three domains of life. *In silico* molecular docking studies predict that citrate will bind not only to bacterial PNPases from *E. coli* and *Streptomyces antibioticus*, but also PNPase from human mitochondria and the structurally and functionally related archaeal exosome complex from *Sulfolobus solfataricus*. Critically, we show experimentally that citrate also inhibits the exoribonuclease activity of bacterial, eukaryotic and archaeal PNPase homologues *in vitro*. Furthermore, bioinformatics data, showing key citrate-binding motifs conserved across a broad range of PNPase homologues, suggests that this regulatory mechanism may be widespread. Overall, our data highlight a communicative link between ribonuclease activity and central metabolism that may have been conserved through the course of evolution.

## INTRODUCTION

Ribonucleases (RNases) are ubiquitous enzymes that play central roles in RNA metabolism. They are required for the control of gene expression, primarily through the degradation of mRNAs, the processing and degradation of regulatory RNAs and for the maturation and quality control of stable RNAs (1). However, they also have the potential to be incredibly destructive and, consequently, their activity is carefully regulated (2).

**Figure 1.**
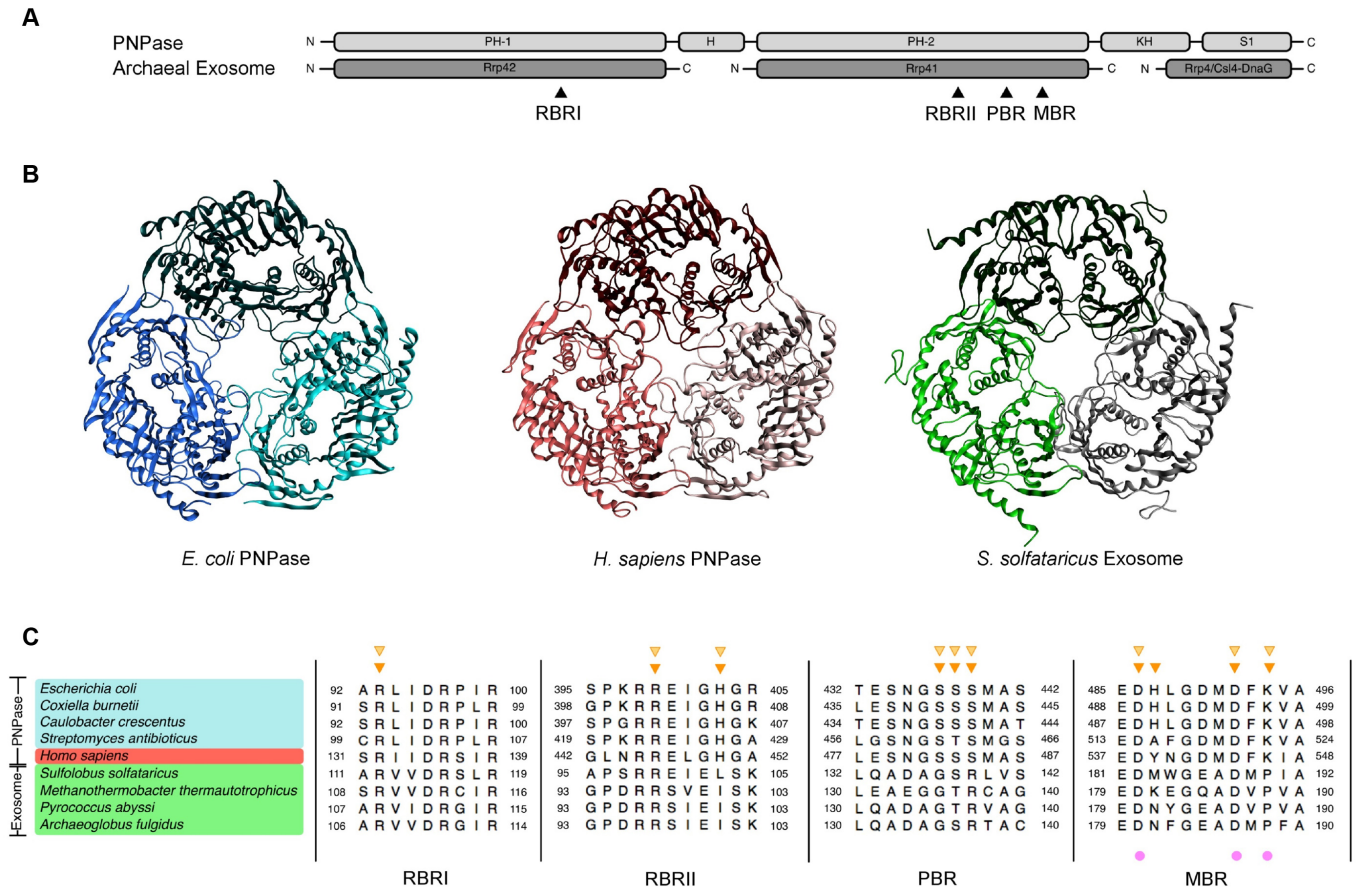
Structural conservation of prokaryotic PNPase, eukaryotic PNPase and the archaeal exosome. (**A**) Schematic representation of the domain organisation of PNPase and the archaeal exosome. The approximate locations of the four motifs (RNA-binding region I (RBRI), RNA-Binding Region II (RBRII), phosphate binding region (PBR) and metal binding region (MBR)) that make up the catalytic centre are indicated with arrows. (**B**) Crystal structures of the PH-hexamer core for *E. coli* PNPase (blue; 3GCM; 9), *H. sapiens* PNPase (red; 3U1K; 13) and *S. solfataricus* Exosome (green; 4BA1; 23). (**C**) Sequence alignment of the four active site motifs (14) in PNPase and the archaeal exosome. Accession numbers are given in Supplementary Table S6. Residues observed to be involved in citrate-binding in the EcPNPase (9,47) and hPNPase (13) crystal structures are indicated above the sequences by dark orange and light orange triangles, respectively. Residues interacting with the catalytic Mg^2+^ ion are indicated below the sequences by magenta spheres.

Polynucleotide phosphorylase (PNPase) is a processive 3΄-5΄ phosphorolytic exoribonuclease that can also catalyse template-independent 5΄-3΄ polymerisation of RNA (3,4). It is widely distributed in bacteria and eukaryotic organelles, but absent from archaea and single-celled eukaryotes such as yeasts (5). PNPases typically contain five domains: two phosphorolysis or RNase PH-like domains (PH-1 and PH-2) separated by an alpha helical domain (H), followed by two RNA-binding domains (KH and S1) (Figure 1A; 5,6). Eukaryotic PNPases also contain an N-terminal signal peptide to target them to the mitochondria or chloroplast (7,8). Structural studies of PNPase from *Escherichia coli* (EcPNPase; Figure 1B; 9,10), *Streptomyces antibioticus* (SanPNPase; 6), *Caulobacter crescentus* (11), *Coxiella burnetii* (12) and *Homo sapiens* (hPNPase; Figure 1B; 13) have revealed a ring-shaped homotrimeric complex, with a core hexamer of PH domains that form a central channel that is able to accommodate single-stranded RNA. The RNA-binding domains are positioned on one side of the PH ring where they can guide RNA into the channel towards the active site (6,10–13). Both structural and mutagenesis approaches have been used to define the architecture of the active site, which is composed of four sub-sites: two RNA-binding regions (RBRI and RBRII), an inorganic phosphate-binding region (PBR) and a divalent metal ion-binding region (MBR) (Figure 1A and C; 14). The catalytic centre is located entirely within the PH-2 domain and comprises the MBR and PBR. Two conserved aspartates, D486 and D492 in EcPNPase, co-ordinate a Mg^2+^ ion and an S[S/T]S motif, S437, S438 and S439 in EcPNPase, is predicted to bind the inorganic phosphate (Figure 1C; 9,14,15). RBRI and RBRII, formed by conserved motifs in the PH-1 domain and PH-2 domain, respectively, contribute conserved arginines, EcPNPase R93, R97, R100, R398 and R399 and a histidine, EcPNPase H403 (Figure 1A and C; 9,14–16).

Although PNPase is not present in archaea (5) there is a structurally and functionally equivalent complex, the exosome (17). The archaeal exosome complex contains five different proteins: Rrp42 (equivalent to PH-1), Rrp41 (equivalent to PH-2) and three RNA-binding proteins named Rrp4 (contains S1 and KH domains), Csl4 (contains S1 and Zn-finger domains) and DnaG (binds polyA) (Figure 1A; 18). Structural studies of exosomes from *Sulfolobus solfataricus* (SsoExosome; Figure 1B; 19–23), *Archaeoglobus fulgidus* (24,25), *Pyrococcus abyssi* (26) and *Methanothermobacter thermautotrophicus* (27) have revealed a similar architecture to PNPase. A trimer of Rrp41/Rrp42 dimers form the ring-shaped PH-domain hexamer (19–27) and three RNA-binding proteins, Rrp4 and/or Csl4-DnaG, assemble on one face of the ring where they channel RNA towards the active site (21–25). The RNA-binding motifs (RBRI and II) and the metal-binding motif (MBR) are conserved between PNPase and the archaeal exosome (Figure 1C), however, the phosphate-binding S[S/T]S motif in PNPase has been replaced by a G[S/T]R motif in the exosome (Figure 1C; 23,27). Exosome complexes are also found in eukaryotes, but, unlike their archaeal counterparts, their PH-core does not possess phosphorolytic activity (28,29). Instead, they rely upon accessory proteins with hydrolytic RNase activity to degrade RNA (29,30).

In terms of physiological function, the most studied PNPase is that from *E. coli*. In this bacterium, PNPase is required for growth at low temperatures (31) and its activity has been implicated in all aspects of RNA metabolism: mRNA turnover (32,33), degradation of small regulatory RNAs (sRNAs; 34,35) and the quality control of stable RNAs (36,37). Not surprisingly, given these diverse roles, EcPNPase activity is tightly controlled at both post-transcriptional and post-translational levels. Post-transcriptionally, the stability of the *pnp* mRNA and, therefore, expression of *pnp* is autoregulated by PNPase in a mechanism involving the endoriboculeases RNase III and RNase E (38,39). EcPNPase can function independently but it is also a component of several multiprotein complexes. It is found in the canonical degradosome along with RNase E, the RNA helicase RhlB and the glycolytic enzyme enolase (40,41), it can form an α_3_β_2_ complex with RhlB (42) and it associates with poly(A) polymerase and the RNA chaperone Hfq (43). These associations with another ribonuclease, a helicase and a polyadenylation complex are likely to assist EcPNPase in its degradative capability. However, localisation to the cytoplasmic membrane as part of the degradosome (40,41,44) would serve to restrict its activity. More recent studies have suggested that there is also a communicative link between the cellular metabolic state and EcPNPase activity. ATP (45) and cyclic di-GMP (46) have been shown to modulate EcPNPase activity *in vitro* and our laboratory has demonstrated that the Krebs cycle intermediate, citrate, regulates EcPNPase exoribonuclease activity both *in vitro* and *in vivo* at physiological concentrations of citrate (47). Furthermore, we have previously shown that the status of the cellular metabolome is dependent upon EcPNPase activity (47).

In the current study, we use a combination of bioinformatics and *in silico* molecular docking approaches to show that citrate is likely to bind to PNPase and related exoribonucleolytic proteins from diverse bacterial species, eukaryotic organelles and archaea. Furthermore, we demonstrate experimentally that in addition to inhibiting *E. coli* PNPase, citrate also inhibits the exoribonuclease activity of PNPases from cyanobacteria and human mitochondria, and the archaeal exosome *in vitro*. It is therefore possible that the citrate-mediated regulatory mechanism previously identified in EcPNPase (47) may be conserved across all three domains of life.

## MATERIALS AND METHODS

### Bioinformatics

Protein sequences were extracted from the NCBI RefSeq database (Genbank) using polynucleotide phosphorylase, for prokaryotic and eukaryotic sequences and Rrp41/Rrp42, for archaeal sequences, as search terms. Multiple sequence alignments were built using MAFFT v.7 (48) using the G-INS-i iterative refinement strategy and default parameters: BLOSUM62 and a gap penalty of 1.53. In order to generate the sequence logos, the alignments were manually curated to remove duplicate and partial entries. Close homologues were omitted from the alignments using Jalview (49) to filter out redundancy at 95% sequence identity. The remaining aligned sequences were trimmed to the ‘PH-core’ boundaries using the known sequence/structures as a guide (EcPNPase residues 1–549, hPNPase 1–601, SsoExosome 1–532). The consensus sequences for the RBRI, RBRII, PBR and MBR motifs (14) were identified using this final set of sequences (3509 prokaryote sequences, 252 eukaryote sequences and 69 archaeal sequences; accession numbers are given in Supplementary Tables S1–S4) and visualised as sequence logos with the probability score on the Y-axis using Weblogo3 (50).

### *In silico* molecular docking

Structures for EcPNPase (3GCM, chain A; 9), hPNPase (3U1K, chain A; 13), SanPNPase (1E3P, sole chain; 6) and SsoExosome (4BA1, chain A; 23) were opened in the program MOE (Molecular Operating Environment, 2013.08; Chemical Computing Group Inc., 1010 Sherbrooke St. West, Suite #910, Montreal, QC, Canada, H3A 2R7). The chains that were selected for docking studies were chosen based on the quality of the structure at the enzyme's active site, i.e. the expected citrate-binding site. The required catalytic Mg^2+^ ion was placed in the hPNPase, SanPNPase and SsoExosome structures based on its position in EcPNPase. The structures were then submitted to a preparation step using the LigX interface which included structure preparation, protonation and energy minimisation. Energy minimisation was carried out using the conjugate gradient method with a cutoff distance of 6 Å, a convergence criterion of 0.01 kcal/mol and using the Amber12:EHT force field that is specifically parameterised for both proteins and small molecules (51,52). Putative ligand-binding sites were identified using the MOE Alpha Site Finder panel, which generates hydrophobic and hydrophilic α-spheres to represent locations of tight atom packing. The appropriate ligand-binding site was then selected using the current knowledge of the expected citrate-binding sites for each protein and defined by the placement of dummy atoms. The structure of the citrate ion was obtained from the ZINC database (53; ZINC ID: 00895081) and saved in a biologically relevant conformation. The Docking module of MOE was used to dock the citrate ion (the ligand) into each of the fixed protein structures (the receptors) using ‘Triangle Matcher’ placement methodology and 300 placement poses. The lowest-energy 30 unique receptor-ligand complexes were identified and scored according to the London dG scoring function (Molecular Operating Environment), which estimates the binding free energy of the ligand. The receptor-ligand complexes were then submitted to a forcefield refinement step with no second rescoring, retaining 30 unique ligand-receptor complexes. The docking scores (S values) for these 30 unique receptor ligand complexes were calculated using the Generalized Born solvation model (GB/VI; 54). This calculates the non-bonded interaction energy (van der Waals, Coulomb and GB implicit solvent interaction energies) between the receptor and the ligand complex whilst excluding the self-energies of the individual receptor and ligand atoms. The lowest-energy pose with a single citrate molecule docked was selected and used as the starting structure to dock a second citrate molecule. Protein ligand interaction fingerprints (PLIFs) were generated in the MOE PLIF panel for the ten lowest-energy poses, with both one and two molecules of citrate docked, for proteins from each of the four organisms. The frequency with which an interaction was observed between citrate and a particular amino acid in the docking experiments was calculated from the PLIFs for both one and two molecules of citrate docked and presented as a heat map generated in Plotly (Plotly Technologies Inc., 2015).

### Protein expression and purification

*E. coli* BL21(DE3)pLysS was transformed with the EcPNPase expression vector, pET-Duet1_EcPNPase, obtained from Prof. B. Luisi (University of Cambridge, UK). EcPNPase expression was induced at an OD_600_ of 0.6 with 0.5 mM isopropyl β-D-1-thiogalactopyranoside (IPTG). Induced cells were grown at 20°C for 20 h. EcPNPase was purified as described previously (9).

The coding sequence for hPNPase (GenBank: BC053660.1) was codon-optimised using GeneOptimizer (GeneArt AF, Life Technologies). The resulting gene was synthesised and ligated between the *Nhe*I and *Sal*I sites of pET28b (Novagen) to generate the pET-28b_H_6_-hPNPase expression vector for expression of hPNPase with an N-terminal hexahistidine tag. *E. coli* BL21(DE3)pLysS was transformed with pET-28b_H_6_-hPNPase and hPNPase expression was induced at an OD_600_ of 0.6 with 0.1 mM IPTG. Induced cells were grown at 25°C for 3 h. hPNPase was purified as described previously (13).

The polycistronic gene encoding the Rrp4 (GenBank: 1454999), Rrp41 (GenBank: 1454998), Rrp42 (GenBank: 1454997) proteins of the SsoExosome was codon-optimised using GeneOptimizer, synthesised and ligated between the *Nde*I and *Xba*I sites of pETMCN-EAVNH, obtained from Dr C. Romier (University of Strasbourg, France). *E. coli* BL21(DE3)pLysS was transformed with the resulting pETMCN-EAVNH_H_6_-Rrp4-Rrp41_Rrp42 vector for co-expression of the N-terminal hexahistidine-tagged Rrp4 and untagged Rrp41 and Rrp42 SsoExosome components. Co-expression was induced at an OD_600_ of 0.6 with 1 mM IPTG and induced cells were grown at 37°C for 3 h. SsoExosome was purified as described previously (21,22).

The sequences of all expression plasmids were verified by DNA sequencing prior to transformation into the *E. coli* BL21(DE3)pLysS expression strain. Following expression and purification, the identity of the recombinant proteins was confirmed by LC-MS of tryptic digests (performed at the Astbury Centre, University of Leeds, UK).

### Exoribonuclease assays

Assays were carried out in reaction mixtures containing 700 nM 5΄-fluorescein amidite (FAM)-A_20_ RNA substrate (Dharmacon), 240 nM enzyme (EcPNPase, SspPNPase (Sigma), hPNPase or SsoExosome), 15 mM Tris pH 8, 112.5 mM NaCl, 3.75 mM MgCl_2_ and 0.045 mM Na_2_PO_4_. Reactions were performed in the absence and presence of 3.75 mM sodium citrate. This concentration of citrate was chosen to be equimolar to the Mg^2+^ concentration and at approximately the K_d_ for the *E. coli* enzyme (47). Assays were incubated at 37°C for 10 min for EcPNPase and SsoExosome, 30 min for SspPNPase and 60 min for hPNPase, to allow for their different relative activities under the assay conditions used. Reactions were terminated by the addition of 0.1 M ethylenediaminetetraacetic acid and reaction products resolved by denaturing 7.5 M urea 20% polyacrylamide gel electrophoresis. Gels were visualised using a Fujifilm FLA-5000 phosphorimager and quantified by densitometry using ImageJ (Rasband WS, ImageJ, U.S. National Institutes of Health, Bethesda, Maryland, USA, http://imagej.nih.gov/ij/, 1997–2016).

## RESULTS

### *In silico* molecular docking of citrate into PNPase and the archaeal exosome

**Figure 2.**
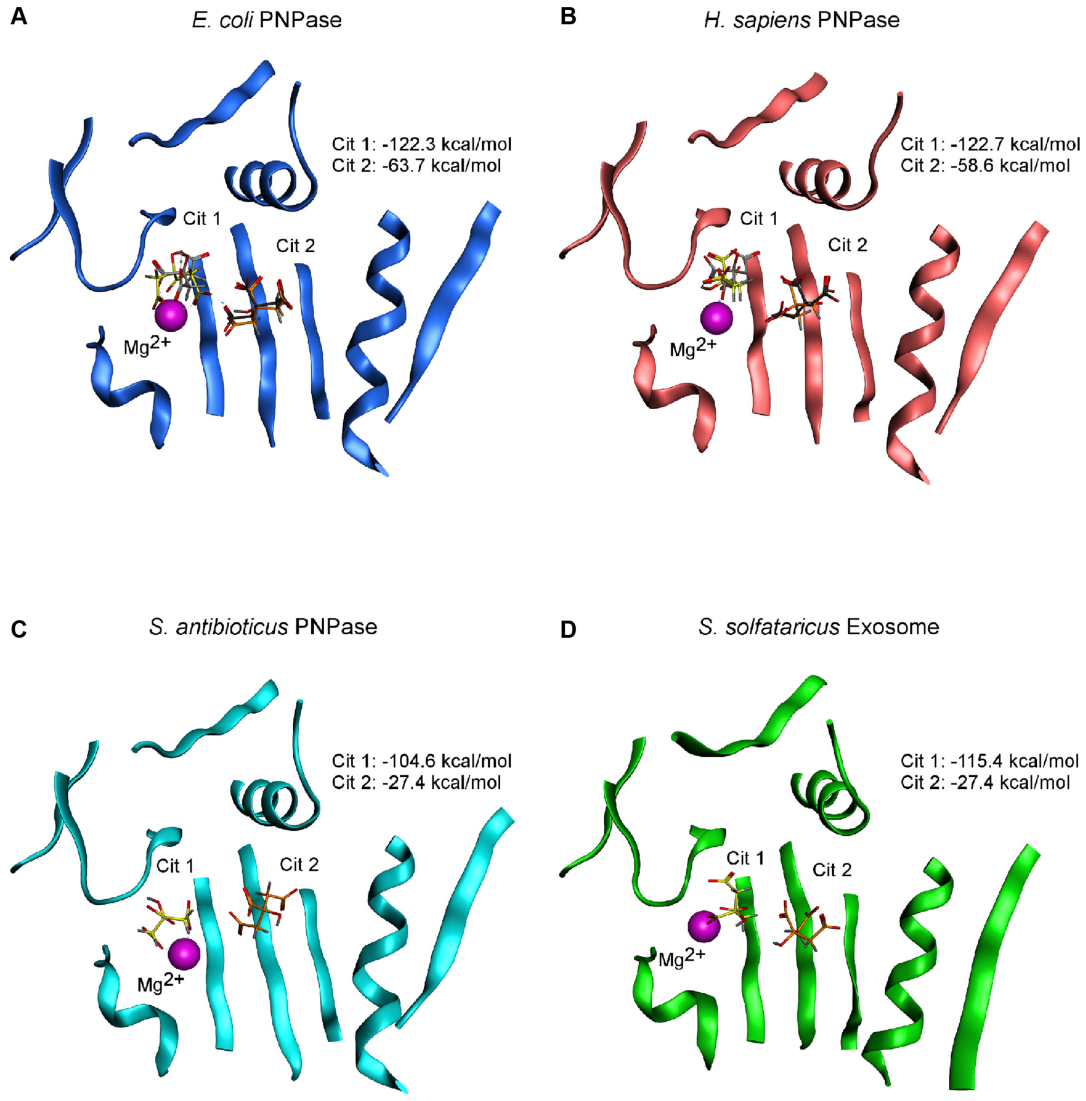
Docking of citrate into the PNPase/archaeal exosome active site. The lowest-energy poses obtained for two citrate ions docked into (**A**) *E. coli* PNPase, (**B**) *H. sapiens* PNPase, (**C**) *S. antibioticus* PNPase and (**D**) *S. solfataricus* Exosome. The protein backbone of the docked structure is shown as ribbons. The catalytic Mg^2+^ ion is shown as a magenta sphere. Docked citrate ions are shown as yellow sticks and orange sticks for the Cit 1 and Cit 2 sites, respectively. For *E. coli* PNPase and *H. sapiens* PNPase, the citrate molecules observed in the crystal structures are shown as light grey sticks and dark grey sticks for the Cit 1 and Cit 2 sites, respectively. The docking score obtained for each of the docked citrate ions is indicated in the top right of each panel.

The first hint that citrate might regulate PNPase came from structural studies of the PNPase PH-hexamer core from *E. coli* (9). In these, 0.2 M ammonium hydrogen citrate had been present in the crystallisation buffer, and the resulting structure contained eight bound citrate molecules (9). Two citrate molecules were located at the active site in all three monomers (9,47). One citrate molecule (Cit 1) occluded the catalytic centre, interacting with S437, S438 and S439 in the PBR and H487, K494 and a Mg^2+^ ion, coordinated by D486 and D492, in the MBR (Figure 1C and 2A; 9,47). The second citrate molecule (Cit 2) mimicked the position of the scissile phosphate in an RNA substrate, interacting with R93 of RBRI and R399 and H403 of RBRII (Figures 1C and 2A; 9,47). Given these binding positions, it was predicted that citrate would inhibit PNPase activity and, critically, exoribonuclease activity was attenuated in the presence of citrate (47). A further two citrate molecules were present in only one of the monomers and were located at the vestigial site (9,47), a site related to the active site by structural duplication but having lost the capacity for catalysis (47). At this site, the citrate molecules were bound by arginines R153, R372, R405 and R409 and this binding was also shown to modulate PNPase activity, most likely through an allosteric mechanism (47).

When the crystal structure of the PNPase core together with the KH domains from *H. sapiens* PNPase was subsequently reported, it too had crystallised in a buffer containing citrate. In this structure, only two molecules of citrate were observed per PNPase monomer, both at the active site (13). There was no evidence of citrate binding to a vestigial site. Alignment of the EcPNPase and hPNPase protein sequences shown in Figure 1C reveals that of the 10 residues identified to be involved in citrate-binding in the EcPNPase active site, 9 are conserved in hPNPase. Only EcPNPase H487 is not conserved; in this position, hPNPase has a tyrosine. Analysis of the interactions between hPNPase and citrate in the crystal structure was performed using the program MOE and revealed that all nine of the conserved residues in hPNPase are involved in citrate-binding whereas the non-conserved tyrosine residue does not interact with citrate (Figures 1C and 2B). This indicates that, in terms of citrate-binding, variability at this position is tolerated. Lin *et al.* (13) did not go on to test the effect of citrate on hPNPase activity. However, based on the sequence homology, it would be predicted that citrate would inhibit hPNPase, similar to EcPNPase.

With mounting evidence that citrate binds to the active site of two distantly-related PNPases, we decided to investigate whether the citrate-binding site is conserved in other PNPases and/or the homologous archaeal exosome. Molecular docking is a valid computational approach to test the potential for ligand binding *in silico* before moving on to more resource-consuming experimental methodologies (55,56).

An alignment of the active site sub-sites for the PNPases and archaeal exosomes for which there is structural information available, highlighting the residues involved in citrate-binding in the EcPNPase and hPNPase crystal structures, is shown in Figure 1C. Comparison of the four bacterial PNPase sequences shows that 8 of the 10 citrate-binding residues, originally identified in EcPNPase, are absolutely conserved. However, SanPNPase contains two substitutions: one at the EcPNPase H487 position that, as noted above, can tolerate variability and a second is a conservative substitution of threonine for serine at the position of EcPNPase S438 (Figure 1C). The residues involved in citrate-binding in hPNPase, as discussed above, are also highly conserved relative to EcPNPase. In contrast, only 4 of the 10 residues involved in citrate-binding in EcPNPase are absolutely conserved in the archaea homologues making it more difficult to predict, based on sequence alone, whether citrate is likely to bind to the exosome (Figure 1C).

These nine protein sequences were similarly analysed to investigate the citrate-binding site observed at the vestigial site of EcPNPase and the resulting alignment of the three citrate-binding motifs is shown in Supplementary Figure S1. The evidence for conservation of citrate-binding at this site is much weaker than for the active site. None of the four residues identified to bind citrate at the EcPNPase vestigial site are absolutely conserved (Supplementary Figure S1) and citrate was not bound at this location in the hPNPase crystal structure (13). Taking both of these observations into consideration, citrate-binding to the vestigial site was not investigated further.

Based on these initial sequence comparisons, structures for EcPNPase and SanPNPase were selected as bacterial representatives for molecular docking studies. The structure for hPNPase was selected as the only structure available for a eukaryotic PNPase, and the SsoExosome structure was selected as the most characterised of the archaeal exosomes.

**Figure 3.**
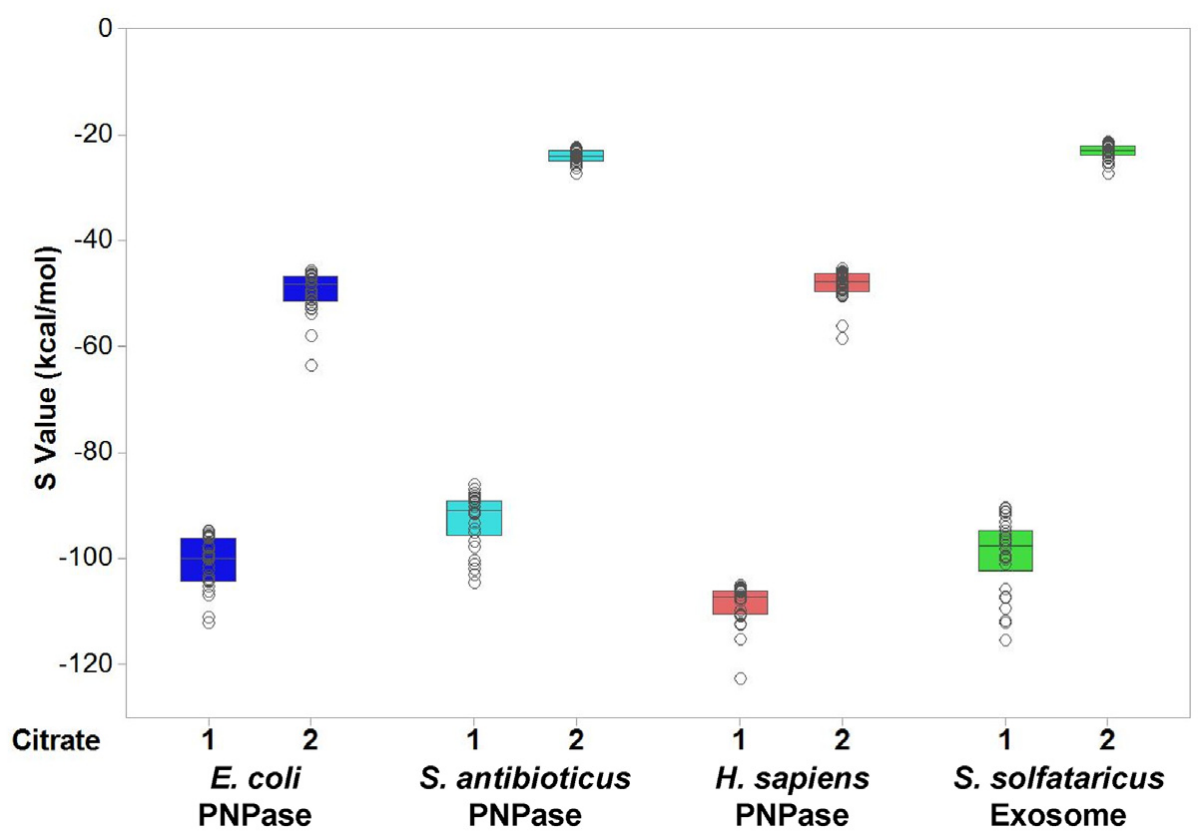
Docking scores for citrate docked into PNPase/the archaeal exosome. A box plot of the molecular operating environment (MOE) docking scores for two citrate ions docked into the active site of *E. coli* PNPase, *S. antibioticus* PNPase, *H. sapiens* PNPase and *S. solfataricus* Exosome. Individual data points are shown as grey circles with the coloured boxes representing the interquartile range and the horizontal line within the box indicating the median docking score. The Citrate 1 docking scores are for 30 unique poses obtained when a single citrate ion was docked into the protein structure. The Citrate 2 docking scores are for 30 unique poses obtained when a second citrate ion was docked into the lowest energy pose obtained with one citrate molecule already docked.

In order to validate the *in silico* approach, we first decided to re-dock citrate into the structures of EcPNPase and hPNPase, since structural studies had already reported that both of these proteins are capable of binding two molecules of citrate at their active site (9,13,47). The structures for EcPNPase and hPNPase were prepared, including placement of the catalytic Mg^2+^ ion and energy minimisation, in MOE. Root mean square deviations calculated between the original crystal structures and the structures prepared for docking are presented in Supplementary Table S5 and indicate that this energy minimisation process did not significantly alter either protein structure. Putative ligand-binding sites were then identified using MOE and defined by the placement of dummy atoms. Encouragingly, potential ligand-binding sites were identified at the active site of both EcPNPase and hPNPase, the known citrate-binding sites. A citrate ion was then docked into each of the structures and, as shown in Figure 2A and B, it docked at the active site (yellow sticks) at the position of Cit 1 in the crystal structures (light grey sticks). The lowest-energy 30 unique poses were retained for each docking calculation and they all placed the citrate ion in this location, albeit in slightly different orientations. The docking scores (S values) for all 30 poses are summarised in the box plot in Figure 3. Due to limitations of the docking software, only a single citrate ion could be docked into each structure in each experiment. As two molecules of citrate bound to the active site of EcPNPase and hPNPase in the crystal structures, we also wanted to explore the potential for a second citrate ion to bind. In order to do this, we used the lowest-energy pose with one citrate ion docked as the starting point for a second round of docking. The second citrate ion docked (Figure 2A and B, orange sticks) at the equivalent location of Cit 2 observed in the crystal structures (Figure 2A and B, dark grey sticks). However, as shown in Figure 3, the S values for this second docked citrate ion were higher (less favourable) than those obtained for Cit 1 for both EcPNPase and hPNPase. Overall, these results are consistent with the conclusion that the *in silico* molecular docking protocol successfully identifies the citrate-binding sites in both EcPNPase and hPNPase.

Having demonstrated the validity of our approach, we next wanted to try docking citrate into the structure of a PNPase that has not previously been shown to bind citrate. We decided to start with the most divergent PNPase for which structural information is available. *E. coli, C. crescentus* and *C. burnetii* all belong to the phylum proteobacteria and all 10 of the residues involved in citrate-binding in EcPNPase are conserved in the PNPases from the other two bacteria. In contrast, *S. antibioticus* belongs to the phylum actinobacteria and, as discussed above, 8 of the 10 residues involved in citrate-binding in EcPNPase are conserved. The two differences are an alanine for histidine substitution at the variability-tolerant EcPNPase H487 position and a conservative substitution of threonine for serine at the equivalent position to EcPNPase S438 (Figure 1C). Thus, based on sequence, it would be predicted that citrate would bind to SanPNPase. As shown in Figure 2C, two citrate ions docked into the active site of SanPNPase. S values obtained for the docking at the Cit 1 site were comparable to those obtained for EcPNPase and hPNPase (Figure 3). However, for the Cit 2 site, the docking scores obtained were significantly higher than for either of the other two PNPases (Figure 3).

The results obtained so far suggest that citrate-binding could be a common property of PNPases. The next step was to investigate the potential for citrate-binding to the more distantly related archaeal exosome homologues. Similarly, to the three PNPases, two citrate ions docked into the active site of the SsoExosome (Figure 2D). The docking scores for the Cit 1 site were similar to those obtained for all three PNPases while the S values for the Cit 2 site were more similar to those obtained for SanPNPase (Figure 3).

**Figure 4.**
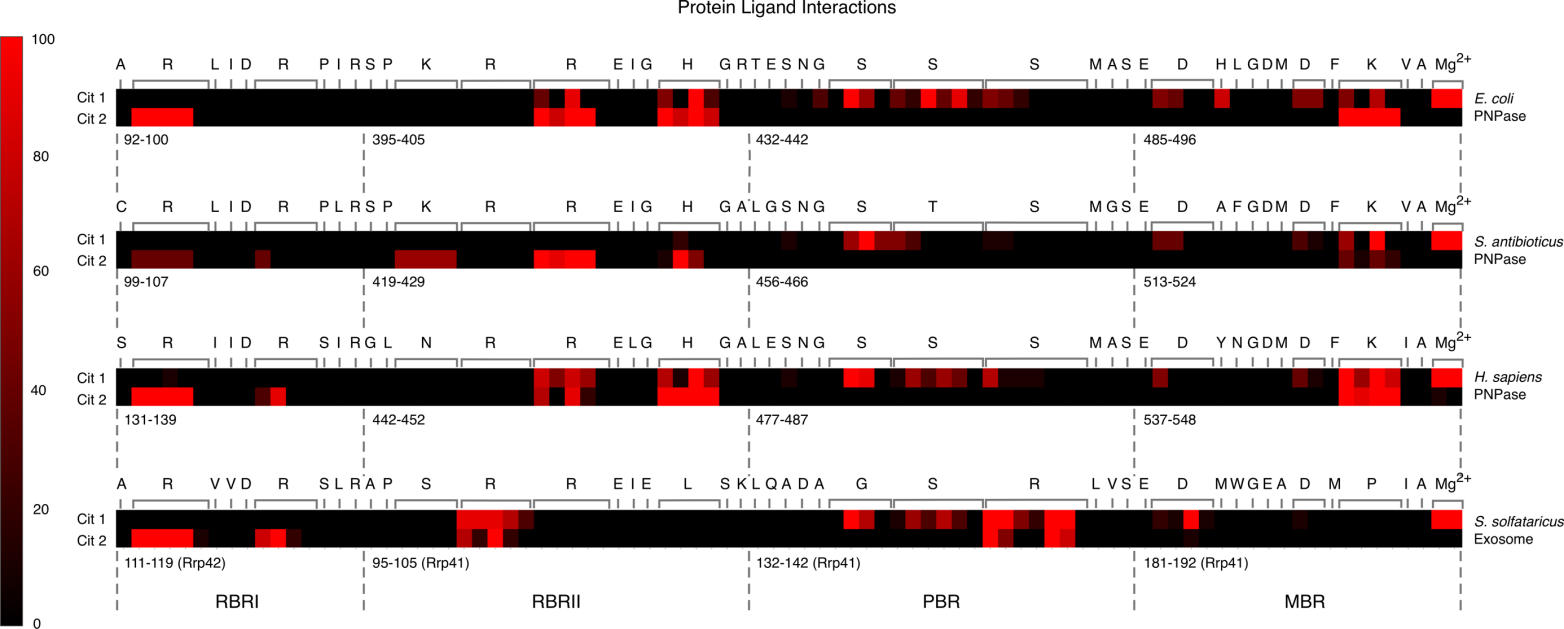
Interactions observed between PNPase/the archaeal exosome and docked citrate. Heat maps summarising the protein ligand interaction fingerprints (PLIFs) generated for the ten lowest-energy poses obtained when two citrate ions were docked into the active site of *E. coli* PNPase, *S. antibioticus* PNPase, *H. sapiens* PNPase and *S. solfataricus* Exosome.

To analyse the docking results further and compare the amino acids predicted to mediate citrate-binding in the three PNPases and archaeal exosome, PLIFs were generated for the ten lowest-energy poses with both one and two citrate ions docked. PLIFs are effectively a barcode for a particular protein:ligand complex, classifying interactions according to whether the interaction is mediated through the main chain or side chain, whether it is polar, non-polar or a hydrogen bond and whether the interaction is strong or weak (57). The frequency with which an interaction was predicted between citrate and a particular amino acid in the docking experiments was calculated from the PLIFs and is summarised in the heat maps presented in Figure 4. Based on the docking experiments, similar interactions are predicted between citrate and the four putative citrate-binding residues that are conserved in EcPNPase, SanPNPase, hPNPase and SsoExosome (EcPNPase positions R93 in RBRI, R399 in RBRII and D486 and D492, via the Mg^2+^ ion in the MBR) in all four complexes (Figure 4). The predicted interactions between docked citrate and the two amino acids that are conserved only in the three PNPase proteins (EcPNPase positions H403 in RBRII and K494 in the MBR) are comparable for the PNPases but there is no equivalent interaction predicted between citrate and the amino acids that are substituted at these positions in SsoExosome (L and P, respectively; Figure 4). Neither sequence nor the predicted interaction is conserved at the position of EcPNPase H487 in the MBR (Figure 4). At the EcPNPase PBR, interactions are frequently predicted between citrate and S437 and S438 with interactions between citrate and S439 predicted less often. The amino acid sequence and pattern of predicted interactions at the PBR is conserved in hPNPase (Figure 4). However, the substitution of threonine for serine at the equivalent position to EcPNPase S438 in SanPNPase appears to have altered the predicted interactions between citrate and the positions equivalent to EcPNPase S438 and EcPNPase S439. In the SsoExosome, EcPNPase positions S437, S438 and S439 have been substituted for G, S and R, respectively (Figure 1C). Despite this sequence variation, significant interactions are predicted between citrate and these three positions in the SsoExosome (Figure 4).

### Inhibition of PNPase and Exosome exoribonuclease activity by citrate

The *in silico* studies suggest that citrate-binding at the enzyme's active site is evolutionarily conserved in bacterial and eukaryotic PNPases and the archaeal exosome. For EcPNPase, this binding has been shown to inhibit exoribonuclease activity providing a mechanism for the fine-tuning of EcPNPase activity (47). We next wanted to experimentally determine whether citrate is also capable of inhibiting other PNPases and/or the archaeal exosome.

**Figure 5.**
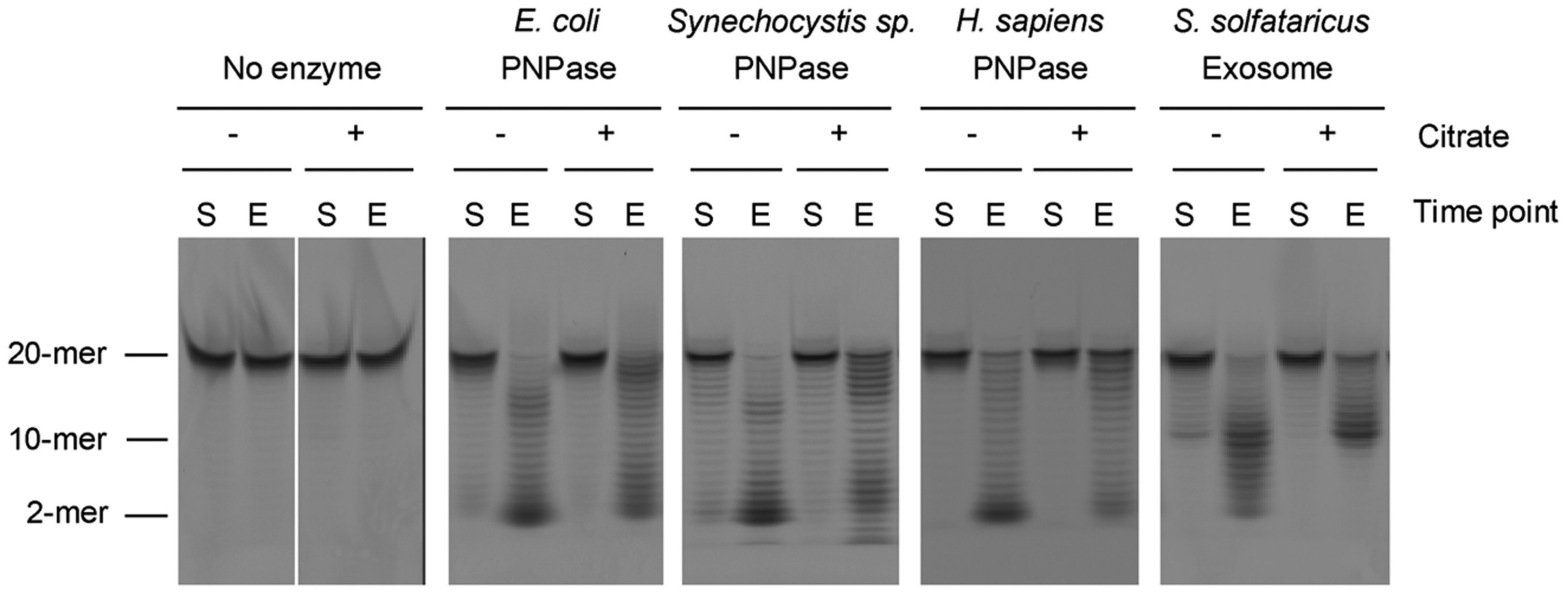
Inhibition of PNPase/the archaeal exosome exoribonuclease activity by citrate. Representative denaturing polyacrylamide gels for end-point exoribonuclease assays carried out using 240 nM *E. coli* PNPase (10-min reaction duration), *Synechocystis sp*. PNPase (30 min), *H. sapiens* PNPase (60 min) or *S. solfataricus* Exosome (10 min) and 700 nM of a 5΄-FAM-A_20_ RNA substrate. The absence or presence of 3.75 mM citrate during the assay is indicated by a − or + sign above the lane, respectively. Lanes labelled S and E indicate samples taken at the start and end of the reaction, respectively.

EcPNPase, hPNPase and the SsoExosome were expressed in *E. coli* and purified to homogeneity and recombinant *Synechocystis sp*. PNPase (SspPNPase) was obtained from Sigma. SspPNPase was used in place of SanPNPase as both have the same threonine to serine substitution at position 438 in the PBR region (Supplementary Figure S2). Exoribonuclease assays using a 5΄-fluorescently labelled A_20_ oligoribonucleotide substrate were carried out for each enzyme/complex in the presence and absence of citrate and analysed by denaturing polyacrylamide gel electrophoresis. An end-point assay performed under similar experimental conditions was selected as the most direct way to compare all four PNPase homologues. Representative gels for this assay are shown in Figure 5. Comparison of the end-point lanes for assays performed in the presence of citrate with those performed in its absence show clear differences for EcPNPase, SspPNPase, hPNPase and SsoExosome. In general, in the presence of citrate, more of the full-length oligoribonucleotide substrate remains at the end of the assay and less of the substrate is fully converted to limit products (Figure 5). There are also some differences in the observed stalling positions in the presence of citrate (Figure 5). This is most striking for the SsoExosome. SsoExosome stalls on oligoribonucleotides around 10 nucleotides in length in the absence of citrate but completely stops at this point in the presence of citrate (Figure 5). Stalling at similar oligoribonucleotide lengths has been previously reported for the *A. fulgidus* and *P. abyssi* exosomes when these short oligoribonucleotides are no longer long enough to bind to both the active site and the entrance to the channel (25,26). Citrate-binding to the active site of the SsoExosome could occlude the only RNA-binding site possible for short oligoribonucleotides and may explain our observation of more of the stalled product.

In an attempt to quantify the effect of citrate upon exoribonuclease activity, the percentage of full-length RNA substrate remaining at the end of the assay was determined for each enzyme/complex in the absence and presence of citrate (Table 1). Given that citrate appears to alter stalling positions, we reasoned that this would provide the fairest comparison across all three of the PNPases and the SsoExosome. EcPNPase activity was clearly inhibited by citrate, with ∼2.5-fold more full-length substrate remaining at the end of the assay in the presence of citrate than in its absence (Figure 5; Table 1). This level of inhibition is consistent with that observed previously for EcPNPase at this citrate concentration (47) and suggests that attenuation of exoribonuclease activity rather than complete inhibition is likely to occur *in vivo*. Citrate also inhibited the exoribonuclease activity of SspPNPase, hPNPase and SsoExosome to a comparable degree as EcPNPase (Figure 5; Table 1). These results clearly demonstrate that citrate can both bind to and inhibit these enzymes *in vitro*.

**Table 1.** Inhibition of PNPase/archaeal exosome exoribonuclease activity by citrate

Enzyme	Percentage substrate remaining at assay end-point	
	− citrate	+ citrate
*E. coli* PNPase	2.2 ± 0.4	5.6 ± 1.5
*Synechocystis sp*. PNPase	2.2 ± 0.2	6.9 ± 2.2
*H. sapiens* PNPase	8.4 ± 2.8	22.8 ± 4.8
*S. solfataricus* Exosome	4.0 ± 0.5	13.6 ± 0.5

Values are the mean from at least three experimental repeats and the errors reported are the standard deviation.

### Sequence analysis of the citrate-binding site

**Figure 6.**
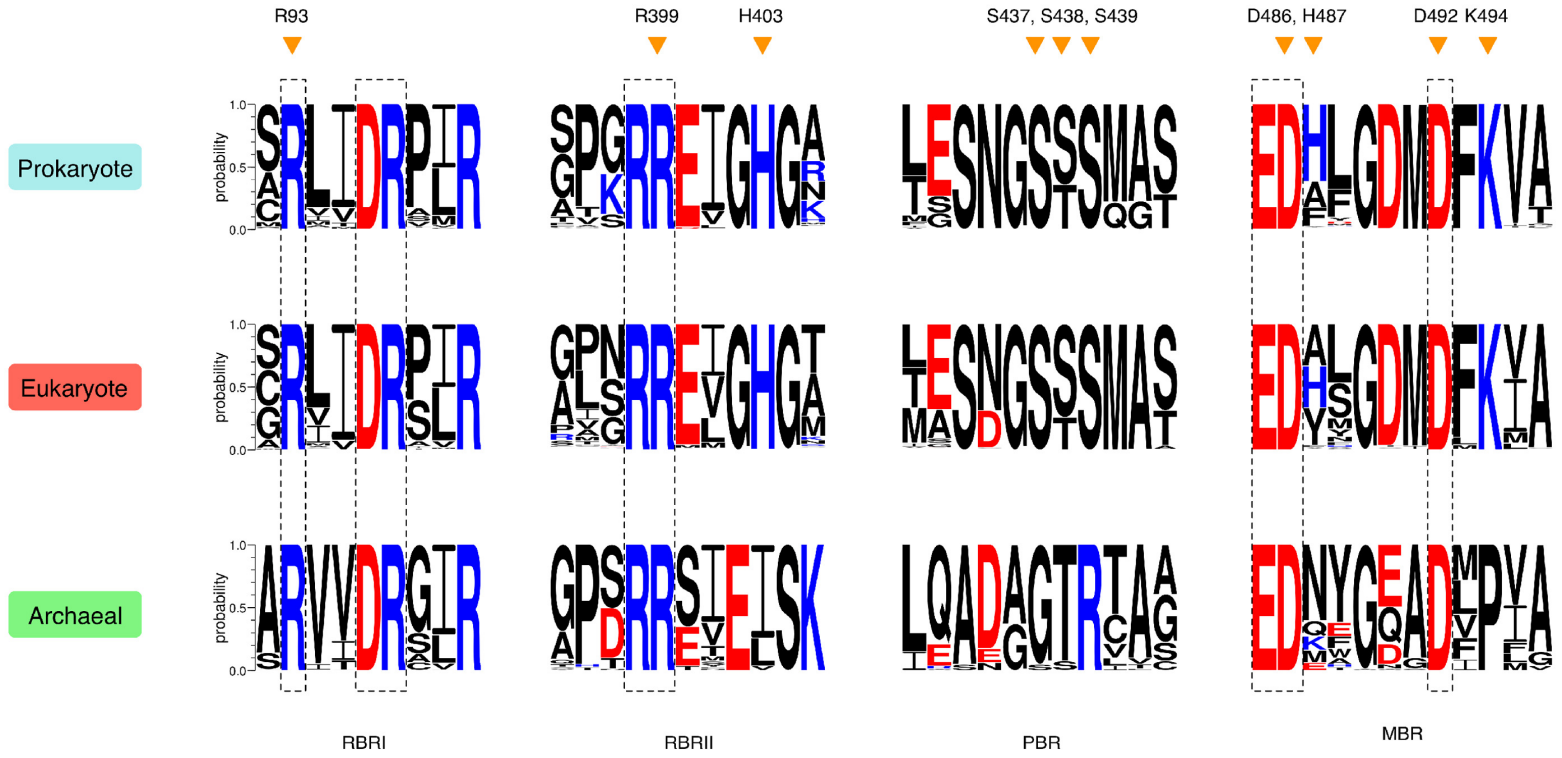
Sequence conservation of the citrate-binding site in prokaryotes, eukaryotes and archaea. Sequence logos for the four motifs (labelled below the sequence logos) comprising the active site/citrate-binding site in prokaryotic and eukaryotic PNPases and the archaeal exosome. Acidic residues are red and basic residues are blue. Residues that are absolutely conserved are indicated by dashed boxes. Residues initially identified to be involved in citrate-binding in *E. coli* PNPase are indicated by orange triangles above the sequence logos together with the residue numbering for *E. coli* PNPase.

The *in silico* and *in vitro* results so far suggest that citrate-mediated inhibition of PNPase and exosome activity occurs in all three domains of life. To assess how widely this inhibitory mechanism is likely to be conserved we carried out sequence analysis of 3509 prokaryotic PNPase, 252 eukaryotic PNPase and 69 Rrp41/Rrp42 exosome protein sequences. The resulting sequence logos (50) for the four active site sub-sites that we have demonstrated are involved in citrate-binding are shown in Figure 6. Of the 10 residues that were originally identified to be involved in citrate-binding in EcPNPase, 8 are absolutely conserved in all of the bacteria and eukaryotes examined (Figure 6). Only positions corresponding to EcPNPase S438 in the PBR and H487 in the MBR show variation. However, the docking and activity results for EcPNPase, SanPNPase/SppPNPase and hPNPase indicate that the sequence variation at these positions is not critical for citrate-binding or citrate-mediated inhibition. The sequence logos for the exosomes (Figure 6) show that four of the residues involved in citrate-binding in EcPNPase are absolutely conserved (R93 in RBRI, R399 in RBRII and D486 and D492 in the MBR). As for the PNPases, variation was observed at the non-critical EcPNPase H487 position in the MBR and also in the EcPNPase H403 position in RBRII and the EcPNPase K494 position in the MBR. Most strikingly, the S(S/T)S motif in the PBR of PNPases appears to have been replaced by a G(T/S)R motif in the archaeal exosomes. However, we have shown for the SsoExosome that citrate has the potential to bind to this motif and, despite the overall sequence divergence, the SsoExosome is inhibited by citrate *in vitro*. Taken altogether these results suggest that all of the PNPases and archaeal exosomes examined are likely to bind to and be inhibited by citrate.

## DISCUSSION

Small molecule metabolites, as *trans*-acting factors, are in a unique position to directly link the cellular metabolic status of a cell and RNase activity. There is a growing body of evidence that such a feedback mechanism is employed in bacteria. Firstly, associations between metabolic enzymes and RNases have now been identified in multi-protein complexes in a number of organisms. Twenty years ago the glycolytic enzyme enolase was shown to be a canonical component of the *E. coli* degradosome (58,59), and recently it has been demonstrated that the *C. crescentus* degradosome contains the Krebs cycle enzyme aconitase (60). Degradosome-like complexes also appear to be present in *Bacillus subtilis* (61,62) and *Staphylococcus aureus* (63) and contain two glycolytic enzymes (enolase and phosphofructokinase), four RNases (RNase Y, RNase J1, RNase J2 and PNPase) and a helicase (CshA). There are also indications that small molecule metabolites modulate RNase activity. Gmr, a c-di-GMP phosphodiesterase, affects the expression of RNase II in *E. coli* (64) implying that the RNase levels are actually regulated by c-di-GMP. Furthermore, PNPase activity has been shown to be directly affected by ATP (45), c-di-GMP (46) and citrate (47) in *E. coli* and (p)ppGpp in *Streptomyces* (65) and *Nonomuraea sp*. (66). The present study indicates that citrate-mediated inhibition of RNases may be far more widespread than these earlier examples would suggest.

We had previously reported that citrate binds to and inhibits the activity of *E. coli* PNPase (47). The level of inhibition observed at physiological concentrations of citrate suggested that attenuation of exoribonuclease activity, rather than complete inhibition, is likely to occur *in vivo* (47). We now demonstrate, both *in silico* and *in vitro*, that PNPases from other bacterial species (*S. antibioticus* and *Synechocystis* sp.) may also be susceptible to inhibition by citrate, which suggests that this attenuation is commonplace amongst prokaryotes. Furthermore, we also show that the activity of both eukaryotic PNPase from human mitochondria and the archaeal exosome complex from *S. solfataricus* is similarly inhibited by citrate. Finally, bioinformatics data suggest that the citrate binding site is highly conserved; indicating that this mechanism of RNase inhibition may be universally employed across all three domains of life.

The molecular mechanism of citrate-mediated inhibition of PNPase appears to involve two citrate molecules binding to the active site, occluding the catalytic centre and the neighbouring RNA-binding regions (47). In the EcPNPase and hPNPase crystal structures, the citrate at the catalytic centre (Cit 1) is bound by three serines at the PBR that are required to bind the inorganic phosphate nucleophile (9), and a lysine and Mg^2+^ ion, coordinated by two catalytic aspartate residues, at the MBR. The binding of the second citrate (Cit 2) is mediated by two arginines and a histidine that play a role in RNA-binding (Figures 1C, 2A and B). Molecular docking studies similarly predicted that these residues would be involved in citrate-binding in EcPNPase, SanPNPase and hPNPase (Figures 2 and 4). Sequence analysis revealed that for bacterial PNPases and those from eukaryotic organelles, these amino acids are highly conserved (Figures 1C and 6), as might be expected for residues that are also known to be critical for enzyme activity. Therefore, given the involvement of these particular residues in citrate-binding, it is predicted that citrate-mediated inhibition of PNPase activity is widely conserved.

Despite there being two possible citrate-binding sites within the PNPase active site, we noted that in the first round of docking the citrate ion was always placed at the Cit 1 site, suggesting that this site has a higher affinity for citrate than the Cit 2 site. The docking scores obtained for each of the two citrate ions support this assertion (Figure 3). It remains to be determined whether both citrates are required for inhibition. Interestingly, the docking scores for Cit 2 for SanPNPase, which has a substitution of threonine for serine at the equivalent position to EcPNPase S438 (Figure 1C), are much higher than for EcPNPase or hPNPase (Figure 3). Nevertheless, SspPNPase, which also contains the threonine for serine substitution, is still inhibited by citrate (Figure 5).

Sequence conservation of both the active site and, consequently, the putative citrate-binding residues is much weaker in the more distantly related archaeal exosomes (Figures 1C and 6). In particular, the PBR S(S/T)S motif, that is required for phosphate-binding in PNPase (9), has been replaced by an G(T/S)R motif in the exosomes (Figure 6). Despite the sequence variation, this motif has also been shown to bind phosphate (25,26) and we have similarly demonstrated the potential functional equivalence with regard to citrate-binding for the *S. solfataricus* exosome (Figures 2D and 4). As for SanPNPase, the docking scores for Cit 2 for SsoExosome are significantly higher than for EcPNPase and hPNPase (Figure 3). Again, this suggests that sequence variation may affect citrate-binding, although, as for SspPNPase, there is no apparent effect on inhibition (Figure 5).

It remains to be determined whether the citrate-mediated inhibition/attenuation of exoribonuclease activity that we have clearly demonstrated *in vitro*, is utilised as a regulatory strategy *in vivo*. For a metabolite such as citrate to act as a regulator the intracellular concentration of the metabolite/citrate must vary under different physiological conditions and the response elicited must be dose-dependent in this concentration range. For EcPNPase, there is evidence to support citrate-mediated regulation. Intracellular citrate concentrations in *E. coli* depend upon the carbon source and have been reported to be 2 mM for growth on glucose or glycerol and 20 mM for growth on acetate (67). Furthermore, the level of inhibition of EcPNPase by citrate observed *in vitro* correlates with citrate concentration in this range (47). For other organisms, the situation is less clear. There is a lack of metabolomics data reporting intracellular citrate concentration(s) and this information is non-trivial to obtain. Nevertheless, taken together, our results strongly suggest that citrate-mediated inhibition of RNase activity may be a common phenomenon witnessed across all three domains of life.

## Supplementary Material

Supplementary Data
