# Supplementary material for: Inhibition of homologous phosphorolytic ribonucleases by citrate may represent an evolutionarily conserved communicative link between RNA degradation and central metabolism
Source: Nucleic Acids Res. 2017 Mar 3;45(8):4655–66. doi: 10.1093/nar/gkx114 (PMC5416783; doi:10.1093/nar/gkx114)
Supplement: Supplementary Data [file gkx114_Supp.zip › nar-03347-z-2016-File008.docx]

**Supplementary Material**

**Inhibition of homologous phosphorolytic ribonucleases by citrate may represent an evolutionarily conserved communicative link between RNA degradation and central metabolism**

Carlanne M. Stone^1†^, Louise E. Butt^1†^, Joshua C. Bufton^1^, Daniel C. Lourenco^1^, Darren M. Gowers^1^, Andrew R. Pickford^1^, Paul A. Cox^2^, Helen A. Vincent^1^* and Anastasia J. Callaghan^1^*

^1^ School of Biological Sciences and Institute of Biomedical and Biomolecular Sciences, University of Portsmouth, Portsmouth, PO1 2DY, United Kingdom

^2^ School of Pharmacy and Biomedical Sciences and Institute of Biomedical and Biomolecular Sciences, University of Portsmouth, Portsmouth, PO1 2DT, United Kingdom

* To whom correspondence should be addressed. Tel: +44 (0)23 9284 2055; Fax: +44 (0)23 9284 2070; Email: Anastasia.Callaghan@port.ac.uk. Correspondence may also be addressed to Helen A. Vincent. Tel: +44 (0)23 9284 2055; Fax: +44 (0)23 9284 2070; Email: Helen.Vincent@port.ac.uk

^†^The authors wish it to be known that, in their opinion, the first two authors should be regarded as joint First Author

**Supplementary Tables**

**Supplementary Tables 1-4 are supplied in Excel file Supplementary Tables 1-4.**

**Supplementary Table 5. RMSDs of the crystal structures compared to the docking structures.**

| **Enzyme** | **RMSD (Å)** |
| --- | --- |
| *E. coli* PNPase | 0.248 |
| *S. antibioticus* PNPase | 0.257 |
| *H. sapiens* PNPase | 0.247 |
| *S. solfataricus* Exosome | 0.249 |

**Supplementary Table 6. Accession numbers for the protein sequences aligned in Fig. 1c, Supplementary Fig. 1 and Supplementary Fig. 2.**

| **Organism** | **Protein** | **Accession Number** |
| --- | --- | --- |
| *Escherichia coli* | PNPase | WP_060707494.1 |
| *Coxiella burnetii* | PNPase | WP_042526278.1 |
| *Caulobacter crescentus* | PNPase | WP_010917924.1 |
| *Streptomyces antibioticus* | PNPase | GI: 75349253 |
| *Synechocystis sp. PCC6803* | PNPase | WP_010871289 |
| *Homo sapiens* | PNPase | NP_149100.2 |
| *Methanothermobacter thermautotrophicus* | Rrp41  Rrp42 | WP_010876322.1  WP_010876321.1 |
| *­­Pyrococcus abyssi* | Rrp41  Rrp42 | WP_010867734.1  WP_010867735.1 |
| *Archaeoglobus fulgidus* | Rrp41  Rrp42 | WP_010878000.1  WP_010878001.1 |
| *Sulfolobus solfataricus* | Rrp41  Rrp42­ | WP_009991308  WP_009991305.1 |

**Supplementary Figure Legends**

**Supplementary Figure 1. Sequence alignment of the three citrate-binding motifs at the PNPase/archaeal exosome vestigial site.** Accession numbers are given in Supplementary Table 6. Residues observed to interact with citrate in the *E. coli* PNPase (9,47) crystal structure are indicated above the sequences by dark orange triangles.

**Supplementary Figure 2. Sequence alignment of the four active site motifs (defined in Portnoy *et al*., 2008 (14)) in PNPase and the archaeal exosome.** Accession numbers are given in Supplementary Table 6. Residues observed to be involved in citrate-binding in the *E. coli* PNPase (9,47) and *H. sapiens* PNPase (13) crystal structures are indicated above the sequences by dark orange and light orange triangles, respectively. Residues interacting with the catalytic Mg^2+^ ion are indicated below the sequences by magenta spheres.

**Supplementary Figures**

**Supplementary Figure 1**

**
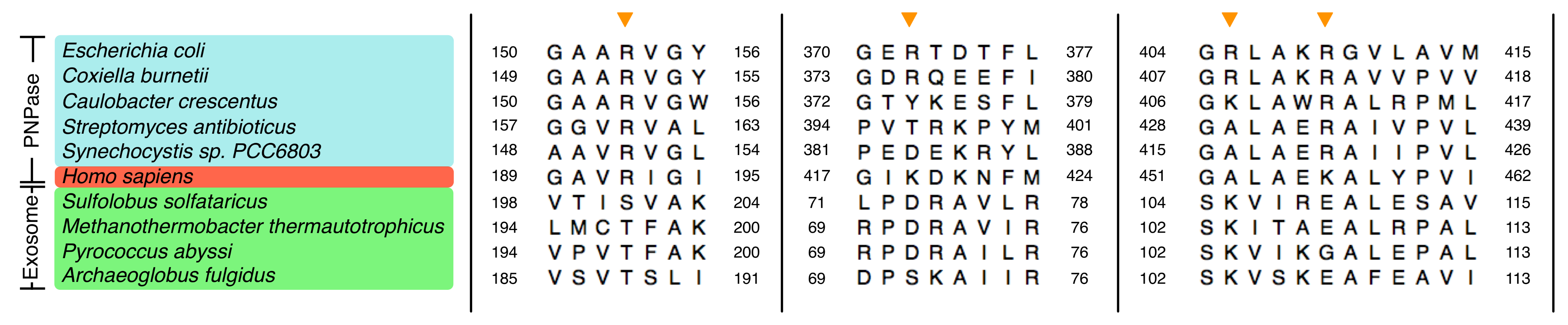
**

**Supplementary Figure 2**

**
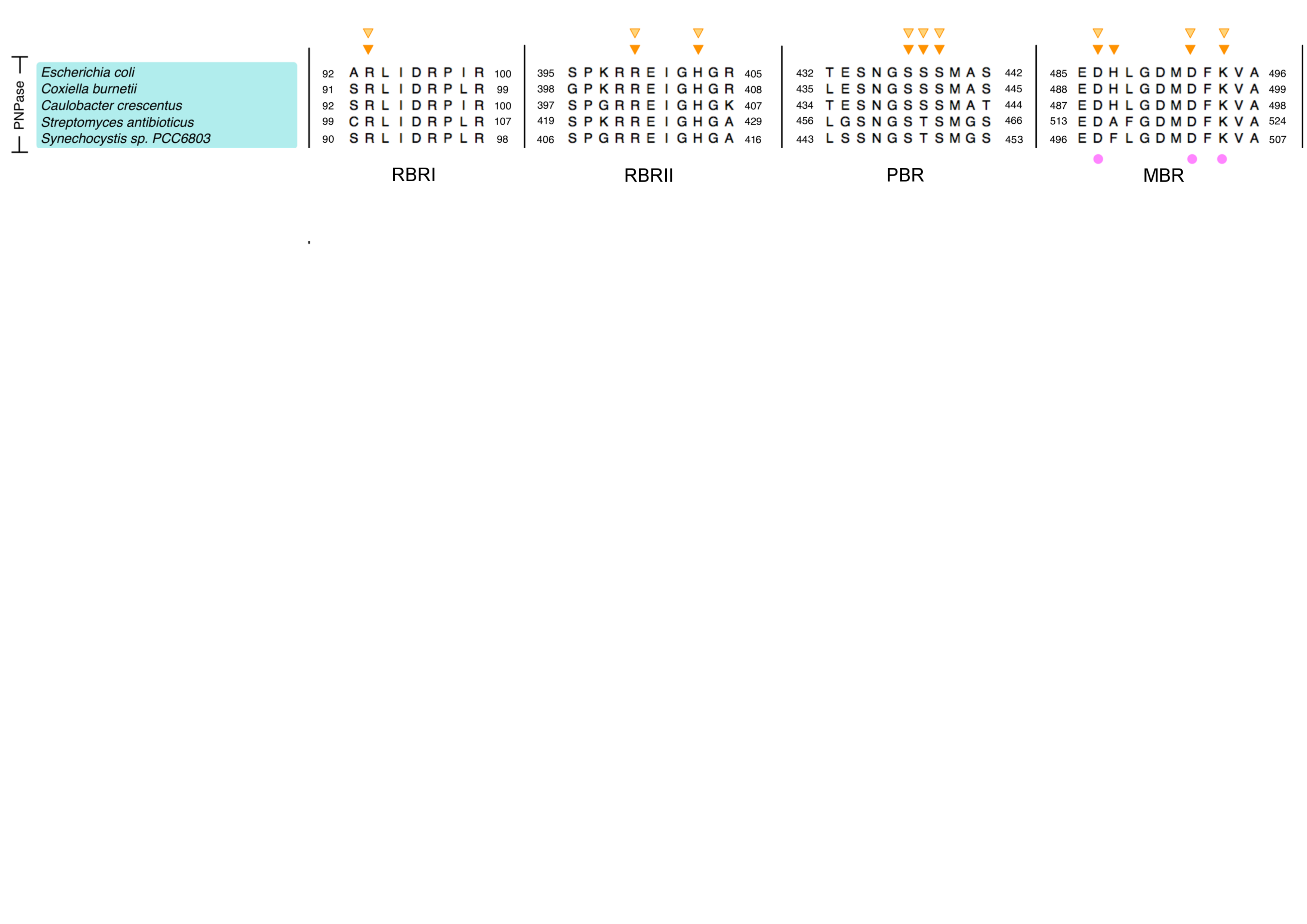
**
